# On the Influence of the Section of Nerves upon the Production of Intestinal Liquids

**Published:** 1868-07-15

**Authors:** M. A. Moreau


### ON THE INFLUENCE OF THE SECTION OF NERVES UPON THE PRODUCTION OF INTESTINAL LIQUIDS.

*Academy of Sciences, Paris, Session of March* 16, M. DELAUNAY, *President.*

BY M. A. MOREAU. PRESENTED BY M. CL. BERNARD.

THE experiment which I am about to describe demonstrates that the section of the nerves supplying the intestine, determines the production of liquids in the intestinal cavity.

Upon a dog of large size, and well fed, but fasting since the previous evening, at least, and whose intestine was consequently empty, I performed, under the influence of chloroform, a section of the abdominal wall, along the linea alba, and laid bare, separating from it the epiploon, a large loop of intestine. I selected upon this loop, with reference to the distribution of the vessels, a length of ten or twenty centimeters (from 4 to 8 inches). I applied two ligatures in such a manner as to have thus enclosed a small loop, which could receive fluids coming neither from above nor from below. I formed, moreover, by the aid of two new ligatures, two other loops, the one above, the other below the first. These three cavities were vacant. I then isolated, with the greatest care, the nerves which distribute themselves upon the middle segment; these nerves proceed in close apposition to the blood vessels, or are placed at some distance from them.

I cut them with caution; then, the intestine and the epiploon being replaced, I sewed up the lips of the wound made in the abdominal wall.

The dog awakening soon afterwards, appeared to be unconscious of the operation which he had sustained. At the end of several hours he was killed, and the abdomen opened. The liquid contained in the enervated segment was collected by puncturing the intestine with the aid of a trocar. The two adjoining segments, unmutilated, were flaccid and empty, contrasting in appearance with the mutilated segment. Their mucous membrane was tight, and even dry to the finger, whilst that of the enervated segment was smooth, humid, and softened by the presence of the newly formed liquid which bathed it.

This liquid contained mucus, white globules, and mucous corpuscles. It was entirely free from red globules, except in the case where the thread which closed the intestines should have cut the sanguineous vessels. I easily avoided this accident, by employing ligatures which do not cut, such, for example, as tubes of caoutchouc of small diameter.

By rest, this liquid deposited mucus, some traces of alimentary matters, and frequent debris of tænia, also mucous corpuscles and leucocytes, the quantity of which offers variations very interesting to study. This liquid, when filtered, is clear, with a slight yellowish tint; its density equals 1.008. It is strongly alkaline, and contains a quantity of carbonate or of bicarbonate, corresponding to 0.2 gr. of anhydrous *Soda* per 100 grammes.

The organic matters furnished a weight of 35 to 45 centigrammes, and the mineral matters a weight of 9 to 9.5 decigrammes per 100 grammes of the liquid. The fixed residuum consisted of alkaline carbonates, chlorides, and a little sulphate and phosphate of lime.

A quantitative analysis of three different portions of the liquid showed that the sodium varied from 34 to 36 per cent., the potassium from 2 to 6, the chlorine from 32 to 45, and the sulphuric acid from 1 to 4. The phosphate of lime weighed during a single analysis formed about two per cent. of the residuum.

Upon the addition to the filtered liquid of *Acetic acid*, in sufficient quantity to neutralize the alkali, there was obtained by ebullition a coagulum whose weight varied from 8 to 10 centigrammes, and which represented also the third or the fourth of the organic matters. Urea was found in the non-coagulated matters; measured by an analysis, it furnished a weight of 16 milligrammes per 100 grammes of the liquid.

The quantity of liquid obtained in a segment of intestine rose to 100 grammes in a dog killed three hours after the operation; and to 225 grammes in another, which I exhibited to the Biological Society, and which had been killed eighteen hours after the operation.

The comparison of this liquid with the intestinal juice, the study of the conditions which cause the variations in the quantities obtained, and other points which presented themselves to my observation deserve, I think, to be developed in another communication.

In brief, the section of the nerves supplying an intestinal loop, determines the production of liquids in that loop. The portion of intestine distant one centimetre higher or lower, remains in complete repose as regards the production of intestinal liquids.

I have made, and am now prosecuting these investigations, in the laboratory of M. Cl. Bernard at the College of France, since the month of May last.

